# Sobriety and alcohol use among rural Alaska Native elders

**DOI:** 10.3402/ijch.v75.30476

**Published:** 2016-02-04

**Authors:** Monica C. Skewes, Jordan P. Lewis

**Affiliations:** 1 Department of Psychology, Montana State University, Bozeman, MT, USA; 2 Indigenous Wellness Research Institute, School of Social Work, University of Washington, Seattle, WA, USA

**Keywords:** CBPR, alcohol research, rural Alaska, Alaska Native health, elders

## Abstract

**Background:**

Although notable health disparities related to alcohol use persist among Alaska Native people living in rural communities, there is a paucity of research examining drinking behaviour in particular segments of this population, including elders. One explanation for this is the distrust of behavioural health research in general and alcohol research in particular following the legacy of the Barrow Alcohol Study, still regarded as a notable example of ethics violations in cross-cultural research.

**Objective:**

The present study reports findings from one of the first research studies asking directly about alcohol abuse among rural Alaska Natives (AN) since the study in Barrow took place in 1979.

**Design:**

We report findings regarding self-reported alcohol use included in an elder needs assessment conducted with 134 Alaska Native elders from 5 rural villages off the road system in Alaska. Data were collected in partnership between academic researchers and community members in accordance with the principles of Community-Based Participatory Research.

**Results:**

Findings showed very high rates of sobriety and low rates of alcohol use, contradicting stereotypes of widespread alcohol abuse among AN. Possible explanations and future research directions are discussed.

**Conclusions:**

This research represents one step forward in mending academic–community relationships in rural Alaska to further research on alcohol use and related health disparities.

The demographic profile of America's older population is projected to change dramatically in the next century. The elderly population in the United States is expected to increase 230% by the year 2050 (1), and the population of ethnic minority elders is expected to grow 510% (2). According to the Administration on Aging, Alaska saw a 52.1% increase in its age 65 and older population between 1999 and 2009 (3). This was the top growth rate for any age group in the US, and more than 3.5 times the national growth rate of 14.6%. The number of Alaska Natives (AN) aged 65 and older is estimated to triple from 6,156 to 19,004 between 2000 and 2030 (3). The growing population of older Americans brings increased need for research to understand factors affecting health, well-being and quality of life.

## Alcohol use and disorders among older adults

Alcohol use disorders are an important health disparity affecting older adults in the US Some older adults engage in frequent binge drinking, defined as 4 or more drinks for a woman or 5 or more drinks for a man on one occasion (4). Rates of alcohol abuse and binge drinking are increasing among older adults across the United States (5) as well as in Alaska (6). People over age 65 binge drink, on average, 5–6 times per month, compared to about 4 times per month for younger adults (4). Furthermore, older adults with alcohol use disorders suffer greater age-specific mortality rates (81.6 deaths per 100,000 for those aged 45–54 and 89.3 deaths per 100,000 for those aged 55–64; 7) across all racial and ethnic groups. Alcohol abuse results in health consequences at any age, but is especially problematic for older adults because of the added health burden of chronic diseases and the possible interactions with prescription drugs commonly prescribed to aging individuals. In addition, addressing substance abuse in later life is challenging because the prevalence of the problem has remained underreported and underdiagnosed, and has not received much attention from treatment providers and researchers (8,9). Providers and patients alike may falsely attribute symptoms of alcohol problems to other symptoms common in elderly populations, such as forgetfulness and falls (10). Providers and members of social support systems often believe that drinking is infrequent among older adults or is one of the only pleasures the elderly are allowed in daily life, resulting in reluctance to screen for problem drinking (10,11). The erroneous assumption that interventions are not worthwhile or effective among older adults also must be challenged (10,11).

Shame surrounding having a substance use problem is another barrier to treatment and health care for older people with alcohol use disorders (12). Many people feel ashamed of having an alcohol problem due to social and cultural stigma surrounding substance abuse and mental health (13,14), and the perception of stigma may be even greater among the elderly who learned about alcoholism during the pre-1950s era of the moral model of addiction (10). Older adults typically view alcohol use and related problems (e.g. depression) to be private matters that one should manage on one's own, resulting in a reluctance to seek treatment (10). Acknowledging that one needs help to change an addictive behaviour like problem drinking may be particularly difficult among those who view seeking treatment as a sign of weakness, and feeling ashamed about needing help for substance abuse or other mental health problems is a known barrier to recovery from addiction problems among people of all ages (15). When alcohol problems are viewed as signs of personal weakness or failure, they are more difficult to talk about and therefore more difficult to diagnose and treat.

## Alcohol-related health disparities among Alaska Natives

AN people experience a wide range of health disparities compared to the general population and evidence higher morbidity and mortality rates than US Whites for 9 of the 10 leading causes of death (i.e. cancer, unintentional injury, suicide, alcohol abuse, chronic obstructive pulmonary disease, cerebrovascular disease, chronic liver disease, pneumonia and influenza, and homicide; 16). According to the Alaska Native Tribal Health Consortium, the AN alcohol-related mortality rate is 16.1 times higher than the rate for Whites in the US, and alcohol is responsible for 14.1% of premature deaths among ANs (16). Alcohol abuse is the leading cause of death for AN men and ranks 6th for AN women (16). Alcohol use disorders are linked to several causes of mortality that are significantly higher in AN compared to White populations, including suicide, homicide and non-intentional injuries (17). ANs also suffer substantially higher rates of cirrhosis of the liver, alcohol-related dementia and accidental death as a result of alcohol use disorders (17). Across age groups, mortality from alcohol use disorders among AN men is 10.9 times higher than among White men, and the mortality rate for AN women is 35.1 times higher than among White women (16). The age-specific mortality rate for alcohol abuse is 36.1 deaths per 100,000 for ANs aged 65 years and older, compared to 4.4 deaths per 100,000 among US Whites of the same age (16). In describing these notable health disparities related to alcohol use, we must be cautious to avoid adding stigma to an already-stigmatized population. The roots of these disparities lie in the broader social and ecological context of colonization and its accompanying racism, disenfranchisement and disempowerment of AN peoples. Health disparities are rooted in social and economic injustices affecting multiple health problems in numerous ethnic minority populations (e.g. 18, 19). Clearly, however, disparities in alcohol abuse and alcohol-related disease affecting AN populations and AN older adults are a priority health concern.

## Present study

The present study reports descriptive and correlational data regarding alcohol use among AN elders residing in remote, rural villages in Alaska. Data were gathered during an elder needs assessment in the Northwest region of Alaska, consisting of Inupiat and Yup'ik Eskimo cultural groups. We conducted this study to understand the health status and concerns of elders in the region, adhering to the principles of Community-Based Participatory Research (CBPR; 20). CBPR is a research framework successfully used with tribal communities as an alternative to mainstream western research approaches (e.g. 21, 22). CBPR involves equitable partnerships between academic and community co-researchers and uses research as a tool of empowerment by focusing on the community's needs, priorities, strengths, knowledge and expertise (20,23). For example, we developed trusting and respectful relationships with community participants prior to proposing data collection of any kind; we focused on an area of concern to the communities (i.e. healthy aging); we assessed strengths and protective factors in addition to risk factors; and we disseminated findings to the communities, securing tribal approval before submitting this manuscript for publication. Given the population data regarding alcohol-related health disparities among elders and among ANs, we investigated self-reported alcohol use as one component of the needs assessment. Although disparities in alcohol abuse and health consequences are notable, very little alcohol research has taken place among ANs due to an unfortunate history of research ethics violations. To understand how sensitive alcohol research is among ANs, some background information is needed.

## Alcohol research among AN

A history of research ethics violations has led to great distrust of research among AN people. In particular, unethical and insensitive research about alcohol use described an AN community as a “society of alcoholics” (24), resulting in widespread stereotyping and stigmatization of AN peoples (25). The Barrow Alcohol Study (24,26) was undertaken in 1979 to understand the relationship between alcohol and accidental death, suicide and violence in rural Alaska. The research was conducted without adequate input from the community and used measures that were not validated by the researchers with the population under investigation. The interpretation of the findings neglected to consider the cultural, social, historical and political context that contributed to drinking among ANs, and findings were vastly overgeneralized (24,26). Moreover, the findings were published in the *New York Times* prior to dissemination to the community, precluding collaborative efforts at interpretation of the data (24). As stated in an academic critique of the study:

The research on alcohol abuse and the news coverage was the most demeaning and reprehensible sham. Instead of using Winchester and Remington rifles to destroy a people and a culture, as with the Indians in the 1880s, they bent words, numbers, and statistics to accomplish what was in effect a social and cultural genocide (24, p. 14).

The Barrow Alcohol Study serves as a cautionary tale for researchers working with cultures outside of their own and illuminates the importance of research participants’ rights and autonomy, the potential negative effects of data misuse, and the consequences of poor conceptualization of research findings (27). The breach of research ethics resulted in many communities being unwilling to participate in research in general and alcohol research specifically, thereby halting scientific progress and efforts to improve public health in rural Alaskan communities. The study still is used as an example of violations of research ethics and illustrates what can result when the values, beliefs, experiences and culture of a community are not given careful and due consideration (27). On the other hand, some positive outcomes did result from the Barrow Alcohol Study, including the development of tribal review boards, regional health corporation ethics committees and increased community engagement throughout the research process – all examples of tribal sovereignty and self-determination.

## Community-based participatory research

As stated previously, CBPR is a collaborative and participatory framework for research involving equitable partnerships between academics and community members (25), and was crucial to the success of this research. To help avoid ethics violations like those of the Barrow Alcohol Study, tribal communities prefer or even mandate CBPR approaches to research. The present study began with building a partnership between researchers from the University of Alaska Fairbanks and community stakeholders in Inupiaq and Yup'ik Eskimo villages participating in a study funded by the Administration on Aging to address nutrition and caregiving needs among older AN adults residing in rural Alaska. Prior to entering the community and recruiting participants, the researchers met with tribal councils and local elder coordinators to discuss and obtain approval for the project. The elder coordinators held positions in each of the participating communities to serve as key support personnel for local elders, assist them with applying for and receiving nutrition and other support services, and advocate for their needs. The research team assisted the coordinators with administering the standardized survey required by the funding agency, which included questions about alcohol use. That the communities approved research including questions about alcohol use is evidence of the strong relationships and trust built between the academic researchers and community partners.

## Northwest region of Alaska

**Fig. 1:**
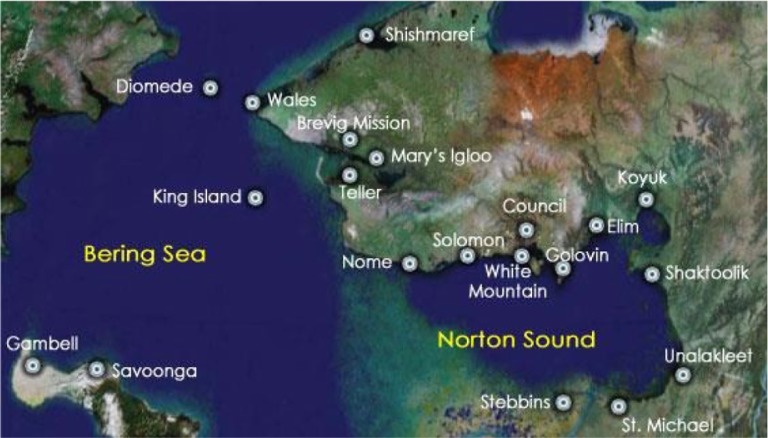
Map of Northwest Alaska Villages.

Understanding the unique geography and cultural context of the region of Alaska from which participants were recruited will help contextualize the findings. The Northwest region has 9,245 residents, of whom 75% are ANs falling into 3 distinct linguistic and cultural groups: Inupiat, Central Yup'ik and Siberian Yup'ik (7). The population of the regional hub community is fairly evenly split between Natives and non-Natives with the populations of the neighbouring villages being almost exclusively AN. Village economies are a hybrid of cash and subsistence and very few jobs are available to their residents, many of whom still live traditional lifestyles, relying on land, river and sea for much of their food (28). See Fig. 1 for a map of the region.

## Method

### Participants

Participants included 134 AN elders residing in remote, rural communities off the road system in Northwest Alaska, which is approximately 32% of the total population of those over age 55 in the region (see Table I). Participants were between 55 and 88 years of age (M= 68.75, SD=8.02) and both men (54.9%, n=73) and women (45.1%, n=60) were represented. Participants identified predominantly as AN (97.7%, n=130) orAmerican Indian (1.5%, n=2) with 2 cases missing ethnicity data. The majority were enrolled members in federally recognized tribes (93.3%, n=125) and spoke a traditional AN language in addition to English (51.5%, n=69).

**Table I:** 2010 Census population data for Norton Sound

Community	Population (all ages)	Population ages 55 +
Village 1	688	145 (21%)
Village 2	368	71 (19%)
Village 3	251	36 (14%)
Village 4	547	84 (15%)
Village 5	297	85 (29%)
Total population	2,151	421 (20%)

Note. Villages in the study are not named to protect community confidentiality.

### Procedure

The research team worked collaboratively with 5 rural villages over 2 years to conduct an elder needs assessment. Before conducting the study, human subjects protection approval was obtained from the IRB at the University of Alaska Fairbanks and the tribal councils of the participating villages. Elder coordinators in each of the participating communities assisted with recruitment of participants and served as gatekeepers and points of contact during the study. Prior to the research team arriving in each community, the elder coordinators provided a list of community members aged 55 years and older who expressed interest in participating in the survey, and scheduled data collection sessions. The research team administered the confidential surveys in interview format in the participants’ homes. The purpose of the survey was to assist tribes, villages and homesteads in creating a record of health and social needs of their elders to be used for tribal planning, long-term care discussions and grant applications. The survey also satisfied the requirement for Title VI nutrition and caregiving grants from the Administration on Aging, which are awarded every 3 years. The research team assisted the communities with managing and analysing the data and submitting the results to secure additional funding for elder nutrition and support services for these 5 communities.

### Measure

The assessment instrument used in this research was the *Identifying Our Needs* survey developed by the National Resource Center on Native American Aging, Center for Rural Health, University of North Dakota (29–31). This standardized instrument asks participants to report on their health status in the following domains: general health and well-being; activities of daily living; mental health; chronic health conditions; vision, hearing and oral health; tobacco and alcohol use; diet, nutrition and exercise; social support, housing and work status; access to health care; use and acceptance of health care services; and unmet needs. The survey instrument was constructed using questions from nationally administered questionnaires so comparisons could be made with data from the general US population. The focus of the present inquiry is on responses to the alcohol use questions.

The assessment of alcohol use began with a description of a standard drink (i.e. 1 can or bottle of beer, 1 glass of wine, 1 wine cooler, a shot of liquor, or a mixed drink) and included the following questions: “How long has it been since you last drank an alcoholic beverage?” and “In the past 30 days, on how many days did you have 5 or more drinks on the same occasion? By ‘occasion,’ we mean at the same time or within a couple hours of each other.” Response options for the first alcohol question included: “within the past 30 days,” “more than 30 days ago but within the past 12 months,” “more than 12 months ago but within the past 3 years,” “more than 3 years ago,” and “I have never had an alcoholic drink in my life.” Response options to the second alcohol question included: “none,” “1 or 2 days,” “3–5 days,” and “6 or more days.” An additional question related to alcohol use was incorporated in the section of the survey about nutrition. Participants were asked to indicate whether they have 3 or more drinks of beer, liquor, or wine almost every day. We examined responses to the alcohol questions in relation to demographic and background items and questions about smoking.

## Results

### Sample characteristics

Most participants were either married (50.7%, n=68) or widowed (26.1%, n=35). Regarding educational attainment, the modal response was having left school prior to beginning high school (30.6%, n=41), although 26.1% (n=35) had graduated high school and 8 participants (6.0%) had earned college degrees. The majority of participants were not employed in the year prior to data collection (69.9%, n=93). Regarding income, 76.6% earned less than $25,000 per year and 58.6% earned less than $15,000 per year. This is notable considering the poverty line in Alaska is $14,580 for a family of one (32), and 85.7% of participants reported household sizes greater than one. The modal response to the question on annual income was under $5,000 per year (21.9%, n=28).

The majority of participants reported residing with family (86.4%, n = 114) and the number of people living in the home ranged from 1 to 13 (M=3.50, SD=2.22).

Health care services were largely provided by the AN health care system, although some participants also received Medicare or Medicaid services. Many participants had previously served active duty in the US military (35.8%, n = 48). Most participants were long-term village residents, with 98.5% having lived in a remote rural Alaskan village for over 5 years and 92.5% having lived at the same address for over 10 years (78.2% over 20 years).

### Sobriety and alcohol use

Contrary to the stereotypes that all Native people have problems with alcohol (i.e. *Firewater Myth*; 33), there were very high rates of sobriety/abstinence reported across the sample. When asked about the length of time since consuming an alcoholic drink, the modal response was *never had an alcoholic drink* (n=38, 30%). Following endorsement of lifetime abstinence, the next most frequently endorsed responses were *more than 3 years ago* (n = 34, 26.5%), *1–3 years ago* (n=22, 17%), *30 days to 1 year ago* (n=18, 14%), and lastly, *within the last month* (n=16, 12.5%). The large majority of the sample had not consumed any alcohol in the year prior to assessment (n=94, 73%). This is in stark contrast to the finding in the general US population that, among adults aged 59–64, over 56% reported consuming some alcohol within the previous month (34). As other research with American Indian and AN populations has noted (35,36), our survey found very high rates of abstinence among rural AN elders.

Low rates of alcohol use also were evidenced by responses to the other questions about alcohol consumption. When asked the number of times that the respondent consumed 5 or more alcoholic drinks in the past month, the vast majority said *none* (n=113, 90%). Eleven participants (9%) reported drinking this quantity on 1–2 days in the past month, and 3 (2%) reported drinking this quantity on 3–5 days in the past month. No participants reported drinking 5 or more drinks in one day in the past month more frequently than 3–5 days. Only 1 participant (0.8%) reported drinking 3 or more drinks almost every day.

Alcohol use among AN elders aged 65 years or older was lower than in the general population. Although our sample included adults aged 55 and over, we examined data separately for those aged 65 and older to allow for comparisons between our sample and other research focused on older adults in the general US population. Among participants aged 65 or older (n=80), 77.9% had not consumed any alcohol in the previous 30 days, compared with 58.8% of elders in the 2012 National Survey of Drug Use and Health (NSDUH; 34, 37). Similarly, a smaller percentage of elders over age 65 in our sample reported past-month binge drinking (defined in the NSDUH as having 5 or more drinks on one day) than those in the general population (5% vs. 8.2%). Finally, only 1 elder over age 65 reported drinking 3 or more drinks daily in the past month (1.3%), compared to 2% of US respondents in the same age group who reported heavy drinking (34,37).

### Local option laws

To determine whether these findings may be influenced by the local option laws instituted in Alaska's rural villages, we examined self-reported drinking rates between people who reside in dry villages (i.e. the importation, sale and possession of alcohol is prohibited by law) and damp villages (i.e. limits on purchase and possession of alcohol are enforced by the community but drinking is not strictly prohibited). Participants reported the name of their home village and we examined the local option laws implemented in those communities via the Alcoholic Beverage Control Board website (38). Most participants lived in dry villages (n=77, 57.5%) but one larger hub community was damp (n= there were no differences between participants from dry and damp communities in either length of time since last drink (t(131)=−0.78, p >0.05) or in the number of heavy drinking episodes in the previous 30 days (t(131)=0.53, p> 0.05). These null findings indicate that the high rates of abstinence and sobriety among this population are not accounted for by local option laws.

### Correlations

Finally, we examined correlations between alcohol use and other variables of interest. Length of time since last alcoholic drink was significantly correlated with several other variables, but frequency of having consumed 5 or more drinks in the past month was associated only with time since last drink (r=−0.59, p <0.001). This indicates that a longer time since one's last drink was associated with fewer instances of heavy drinking in the past month. The finding that no other variables were associated with this indicator of heavy drinking may be due to the very low rates of heavy drinking in this sample. Further, the item asking participants to indicate whether they consume 3 or more drinks on a daily or near-daily basis was not significantly associated with any other variable, also likely due to the very low rates of daily drinking reported.

In addition to being significantly associated with frequency of heavy drinking in the past month, time since last drink also was significantly associated with age (r=0.30, p =0.001). Older people reported a longer period of time since their last drink, suggesting that successful aging involves maturing out of alcohol use (39,40). In rural Alaska, quitting drinking most often occurs without the use of formal treatment (41). Time since last drink was significantly associated with gender, which was coded *1*=*female* and *0*=*male*. The significant positive correlation (r=0.28, p=0.001) indicates that women reported a longer period of time since their last drink than men. Time since last drink was significantly negatively associated with smoking (r=−0.38, p<0.001) and with the number of cigarettes smoked per day (r=−0.22, p <0.05), indicating that people who reported a longer period of time since their last alcoholic drink were less likely to smoke and smoked fewer cigarettes than people who reported drinking more recently. There was no significant correlation between any of the drinking variables and being from a dry vs. damp community.

## Discussion

This research contributes to the literature on alcohol use among older adults in the US by reporting data from rural AN elders, a hard-to-reach and understudied population. Findings from the first phase of this research show low rates of alcohol use and high rates of sobriety among AN elders. Many respondents reported lifelong abstinence from alcohol, and the large majority had not consumed any alcoholic beverages in the previous year. Moreover, 90% of participants reported no episodes of binge drinking or heavy drinking in the past month. These rates are substantially lower than those found in population surveys of elders in the US (34), and suggest that the stereotype of a “society of alcoholics” as described in the Barrow Alcohol Study is unwarranted.

Most importantly, this study has furthered social science research in rural Alaska and demonstrated that alcohol research post-Barrow Alcohol Study can be successful if conducted within the framework of CBPR. This study is among the first to assess alcohol use among AN elders, and took place in full partnership and with full support of the communities that participated. We demonstrate the feasibility of conducting community-based research with ANs from rural areas and provide evidence that research can be done effectively, in a reasonable time frame, and that communities can and do remain involved in interpreting and disseminating findings after data are gathered. For example, the tribal councils approved this manuscript prior to submission with no recommended changes. Adhering to the principles of CBPR and engaging the communities throughout the research process helps to ensure that the findings reflect the local cultures, values and experiences, thereby improving the validity of the findings. It is our hope that the CBPR approach may open the door to further alcohol research in indigenous and rural communities that will not add further stigma or promote stereotyping of Native peoples.

There are several possible explanations for the high rates of abstinence from alcohol among AN elders. First, the high alcohol-related mortality rates among ANs suggest that people with alcohol use disorders die prematurely and do not become elders. It also may be that the prohibition of alcohol in many AN villages, and the accompanying stigma associated with drinking, may influence the willingness of participants to report alcohol use. Yet another possibility is that AN drinkers mature out of alcohol abuse as they age. Cultural and social norms surrounding alcohol use and aging have not been addressed in the literature thus far and must be considered. Although these data are incapable of offering a definitive explanation for the findings, we will address each of these possibilities and suggest future directions for further research.

Health disparities in alcohol-related morbidity and mortality among ANs have been well documented. As stated previously, AN men are 10.9 times more likely to die from an alcohol-related problem than White men, and AN women are 35.1 times more likely to suffer an alcohol-related death than White women (34). Therefore, it is plausible that ANs who drink are less likely to survive to old age. However, this does not explain the high rate of lifetime abstinence evidenced in this study.

It may be that alcohol use was underreported due to its prohibited legal status in dry/local option law villages. Self-reports of illegal behaviour frequently are noted as a limitation of substance abuse research; however, there is substantial research to support the validity of self-reports regarding alcohol use (42,43). Moreover, comparisons between self-reported alcohol use among participants from dry vs. damp communities were not statistically significant. If unwillingness to admit to illegal behaviour were responsible for this finding, we would expect greater abstinence reported among those residing in dry villages. Further, these findings align with other studies showing high rates of lifetime abstinence among American Indian and AN people (44,45). The trust building that took place as part of the CBPR process, the confidentiality assured during the interview sessions, and the rapport established between the researchers and participants likely resulted in honest and valid responses.

It also may be that AN people stop drinking as they age and become respected as elders. Elders are honoured and valued in AN cultures as teachers, leaders and carriers of traditional ecological knowledge. Findings showed that older people reported a longer period of time since their last drink, suggesting that successful aging involves maturing out of alcohol use. In rural Alaska, quitting drinking most often occurs without the use of formal treatment (41). *Natural recovery* is defined as desistence from substance abuse in the absence of formal intervention (46). Although research on natural recovery is limited in AN and rural populations, it appears that many people, especially elders, have recovered from alcohol use disorders on their own (41). More research is needed to understand factors that facilitate or impede resolution of alcohol problems in the absence of treatment, which is often limited in availability and effectiveness in rural communities.

Connected with the notion of maturing out of alcohol problems, age-related reductions in drinking also may be attributed to evolving social roles in the family and community (39,40). In many cultures, older age life stages are associated with changes in social status, norms, expectations and responsibilities (39,47,48). AN elders are expected to serve as role models, help others and maintain positive social relationships (49,50). Many elders feel a responsibility to give back to others, guide younger generations and take on new roles such as caregiver, tribal leader, teacher and bearer of cultural knowledge (41). These roles are often incompatible with alcohol use. As demonstrated in the People Awakening study, many AN people deliberately curtail their alcohol use or stop drinking altogether as a way to live a harmonious and balanced life consistent with AN cultural values (51–53).

### Conclusions and future directions

This research reports selected findings from a quantitative needs assessment of 5 rural AN communities. Strengths of the study include a large sample relative to the size of the population; assessment of a population that is very difficult to reach, both geographically and socially, in which health disparities are well documented but other research is quite limited; use of a standardized survey instrument; and full partnership between academic researchers and community members within a CBPR framework. Findings showed high rates of sobriety and low rates of current and heavy drinking, contrary to existing stereotypes about AN people and alcohol. Perhaps most importantly, this research represents a step forward in alcohol research among AN following the legacy of the Barrow Alcohol Study.

Limitations include the lack of specificity and depth in the assessment instrument regarding alcohol involvement and history. We were not able to determine whether participants had never had trouble with alcohol or if they had recovered from alcohol use disorders. Similarly, for people who may have been in recovery, the instrument did not provide the opportunity to determine whether they received formal treatment or experienced natural recovery. It would be beneficial to prevention and intervention efforts to gather data on drinking motives, outcome expectancies and consequences, as well as in-depth information on readiness to change and motivations for sobriety. Longitudinal studies examining change over time and the process of maturing out of alcohol use among AN elders would fill an important gap in the alcohol literature and in the aging literature. Furthermore, findings from this sample of rural AN elders from the northwestern part of Alaska may not generalize to other populations of AN elders.

Despite these limitations, this research makes a notable contribution to scientific knowledge regarding alcohol use among AN elders. The few questions posed about drinking represent a huge step forward in behavioural health research in this population. The alcohol-related health disparities are serious, and yet progress has been stalled due to understandable distrust of research and researchers. Future research must continue to mend community-academic relationships in Alaska for progress to take place. CBPR is a promising way forward. The trust garnered in the present research must be nurtured to allow for future research and intervention efforts aimed at alleviating health disparities without doing further harm to AN peoples and communities. This study constituted one step toward this end.

## References

[1] Xakellis G, Brangman SA, Hinton WL, Jones VL, Masterman D, Pan CY (2004). Curricular framework: core competences in multicultural geriatric care. J Am Geriatr Soc.

[2] Federal Interagency Forum on Aging-Related Statistics (2000). Older Americans: key indicators of well-being.

[3] Administration on Aging (2010). A profile of older Americans, 2010.

[4] National Institute of Alcohol Abuse and Alcoholism (2015). Drinking levels defined. Bethesda.

[5] Sheridan MK (2012). Binge drinking: seniors engage most often, says study. The Huffington Post [Internet].

[6] Alaska Commission on Aging (2014). Senior snapshot: older Alaskans in 2011/2012.

[7] US Census Bureau (2006). We the people: American Indians and Alaska Natives in the United States.

[8] Dietz TL (2009). Drug and alcohol use among homeless older adults: predictors of reported current and lifetime substance misuse problems in a national sample. J Appl Gerontol.

[9] Outlaw FH, Marquart JM, Roy A, Luellen JK, Moran M, Willis A (2012). Treatment outcomes for older adults who abuse substances. J Appl Gerontol.

[10] Center for Substance Abuse Treatment, Substance Abuse and Mental Health Services Administration (1998). Substance abuse among older adults.

[11] Sorocco K, Ferrell S (1998). Alcohol use among older adults. J Gen Psychol.

[12] Wu LT, Blazer DG (2011). Illicit and nonmedical drug use among older adults: a review. J Aging Health.

[13] Dearing RL, Stuewig J, Tangney JP (2005). On the importance of distinguishing shame from guilt: relations to problematic alcohol and drug use. Addict Behav.

[14] Luoma JB, Kohlenberg BS, Hayes SC, Fletcher L (2012). Slow and steady wins the race: a randomized clinical trial of acceptance and commitment therapy targeting shame in substance use disorders. J Consult Clin Psychol.

[15] Wiechelt S (2007). The specter of shame in substance misuse. Subst Use Misuse.

[16] Alaska Native Tribal Health Consortium (2011). Alaska Native mortality update: 2004–2008.

[17] Alaska Native Tribal Health Consortium (2009). Alaska Native health status report.

[18] Freeman HP (2004). Poverty, culture, and social injustice: determinants of cancer disparities. CA Cancer J Clin.

[19] Braveman PA, Kumanyika S, Fielding J, LaVeist T, Borrell LN, Manderscheid R (2011). Health disparities and health equity: the issue is justice. Am J Public Health.

[20] Israel BA, Schulz AJ, Parker EA, Becker AB (1998). Review of community-based research: assessing partnership approaches to improve public health. Annu Rev Public Health.

[21] LaVeaux D, Christopher S (2009). Contextualizing CBPR: key principles of CBPR meet the Indigenous research context. Pimatisiwin.

[22] Fisher PA, Ball TJ (2003). Tribal participatory research: mechanisms of a collaborative model. Am J Community Psychol.

[23] Wallerstein NB, Duran B (2006). Using community-based participatory research to address health disparities. Health Promot Pract.

[24] Foulks EF (1989). Misalliances in the Barrow Alcohol Study. Am Indian Alsk Native Ment Health Res.

[25] Lewis JP, Boyd K (2012). Determined by the community: CBPR in Alaska Native communities building local control and self-determination. J Indig Res.

[26] Wolf AS (1989). The Barrow studies: an Alaskan's perspective. Am Indian Alsk Native Ment Health Res.

[27] Trimble JE (1989). Malfeasance and foibles of the research sponsor. Damnant quod non intelligent. Am Indian Alsk Native Ment Health Res.

[28] Norton Sound Health Corporation http://www.nortonsoundhealth.org.

[29] Baker-Demaray T, Fasteen D, Gattis M, Ludtke R, McDonald LR, Ruliffson K (2008). Identifying and addressing chronic disease among American Indian elders.

[30] Morin-Carter P, Gray J, Davis J (2008). Identifying our needs: a survey of elders.

[31] Morin-Carter P, Stensland P (2014). Identifying our needs: a survey of elders regional results and trends.

[32] US Department of Health and Human Services (2014). Poverty line.

[33] Coyhis D, White WL (2002). Alcohol problems in native America: changing paradigms and clinical practices. Alcohol Treat Q.

[34] Substance Abuse and Mental Health Services Administration (2015). Results from the 2014 National Survey on Drug Use and Health.

[35] Spicer P (2001). Culture and the restoration of self among former American Indian drinkers. Soc Sci Med.

[36] Whitesell NR, Beals J, Big Crow C, Mitchell CM, Novins DK (2012). Epidemiology and etiology of substance use among American Indians and Alaska Natives: risk, protection, and implications for prevention. Am J Drug Alcohol Abuse.

[37] Substance Abuse and Mental Health Services Administration (2015). State estimates of substance abuse.

[38] State of Alaska (2015). Alcoholic beverage control board.

[39] Quintero G (2000). “The lizard in the green bottle”: “aging out” of problem drinking among Navajo men. Soc Sci Med.

[40] Seale JP, Shellenberger S, Spence J (2006). Alcohol problems among Alaska Natives: lessons from the Inuit. Am Indian Alsk Native Ment Health Res.

[41] Mohatt GV, Rasmus SM, Thomas L, Allen J, Hazel K, Marlatt GA (2007). Risk, resilience, and natural recovery: a model of recovery from alcohol abuse for Alaska Natives. Addiction.

[42] Hesselbrock M, Babor TF, Hesselbrock V, Meyer RE, Workman K (1983). “Never believe an alcoholic?” On the validity of self-report measures of alcohol dependence and related constructs. J Subst Use.

[43] Del Boca FK, Darkes J (2003). The validity of self-reports of alcohol consumption: state of the science and challenges for research. Addiction.

[44] Spicer P, Novins DK, Mitchell CM, Beals J (2003). Aboriginal social organization, contemporary experience and American Indian adolescent alcohol use. J Stud Alcohol Drugs.

[45] Herman-Stahl M, Chong J (2003). Substance abuse prevalence and treatment utilization among American Indians residing on-reservation. Am Indian Alsk Native Ment Health Res.

[46] Walters GD (2000). Spontaneous remission from alcohol, tobacco, and other drug abuse: seeking quantitative answers to qualitative questions. Am J Drug Alcohol Abuse.

[47] Kunitz SJ, Levy JE (1994). Drinking careers: a 25-year follow-up of three Navajo populations.

[48] O'Neill T, Mitchell M (1996). Alcohol use among American Indian adolescents: the role of culture in pathological drinking. Soc Sci Med.

[49] Lewis JP (2011). Successful aging through the eyes of Alaska Native elders. What it means to be an elder in Bristol Bay, AK. Gerontologist.

[50] Moos F, Edwards ED, Edwards ME, Janzen FV, Howell G (1985). Sobriety and American Indian problem drinkers. Alcohol Treat Q.

[51] Hazel KL, Mohatt GV (2001). Cultural and spiritual coping in sobriety: informing substance abuse prevention for Alaska Native communities. J Community Psychol.

[52] Mohatt GV, Rasmus SM, Thomas L, Allen J, Hazel K, Hensel C (2004a). “Tied together like a woven hat”: protective pathways to Alaska Native sobriety. Harm Reduct J.

[53] Mohatt GV, Hazel KL, Allen J, Stachelrodt M, Hensel C, Fath R (2004b). Unheard Alaska: culturally anchored participatory action research on sobriety with Alaska Natives. Am J Community Psychol.

